# A DNA methylation signature to improve survival prediction of gastric cancer

**DOI:** 10.1186/s13148-020-0807-x

**Published:** 2020-01-20

**Authors:** Yaojun Peng, Qiyan Wu, Lingxiong Wang, Huan Wang, Fan Yin

**Affiliations:** 1 College of Graduate, The First Medical Centre, Chinese PLA General Hospital, Beijing, China; 2 Department of Gastroenterology & Hepatology, The First Medical Centre, Chinese PLA General Hospital, Beijing, China; 3 Department of Oncology, The First Medical Centre, Chinese PLA General Hospital, Beijing, China; 4 Department of Scientific Research Administration, The First Medical Centre, Chinese PLA General Hospital, Beijing, China; 5 Department of Oncology, The Second Medical Centre & National Clinical Research Center of Geriatric Disease, Chinese PLA General Hospital, Beijing, China

**Keywords:** Epigenetics, DNA methylation, Gastric cancer, Prognosis, Integrative analysis, TCGA

## Abstract

**Background:**

The current Union International Committee on Cancer or the American Joint Committee on Cancer TNM stage system has shown valuable but insufficient estimation for subsets of gastric cancer and prediction for prognosis patients. Thus, there is an urgent need to identify diagnostic, prognostic, and predictive biomarkers to improve patients’ outcomes. Our aim was to perform an integrative analysis on publicly available datasets to identify epigenetic changes that may play key role in the initiation and progression of gastric cancer, based on which we set to develop a DNA methylation signature to improve survival prediction of gastric cancer.

**Results:**

A total of 340 methylation-related differentially expression genes (mrDEGs) were screened in gastric cancer patients from The Cancer Genome Atlas (TCGA) project. Pathway enrichment analysis revealed that they were involved in the biological process related to initiation and progression of gastric cancer. Based on the mrDEGs identified, we developed a DNA methylation signature consisting of ten gene members (SCNN1B, NFE2L3, CLDN2, RBPMS2, JPH2, GBP6, COL4A5, SMKR1, PPP1R14A, and ARL4D) according to their methylation β value. This innovative DNA methylation signature was associated with cancer recurrence, while it showed independence of cancer recurrence and TNM stage for survival prediction. Combination of this DNA methylation signature and TNM stage improved overall survival prediction in the receiver operating characteristic analysis. We also verified that two individual genes (PPP1R14A and SCNN1B) of the identified prognostic signature were regulated by promoter region methylation in a panel of gastric cell lines.

**Conclusions:**

This study presents a powerful DNA methylation signature by performing analyses integrating multi-source data including transcriptome, methylome, and clinical outcome of gastric cancer patients from TCGA. The identified DNA methylation signature may be used to refine the current prognostic model and facilitate further stratification of patients in the future clinical trials. Further experimental studies are warranted to unveil the regulatory mechanism and functional role of all the individual genes of the DNA methylation signature. Also, clinical investigations in large GC patient cohorts are greatly needed to validate our findings.

## Background

Gastric cancer (GC) is a deadly malignancy, being the fifth most common cancer and the fourth leading cause of cancer death worldwide [1]. The major gastric cancer risk factors include age, gender, race, tobacco use, alcohol consumption, obesity, *Helicobacter pylori* and Epstein-Barr virus infection, gastro-esophageal reflux disease, and family history [2, 3], among which *H*. *pylori* is recognized as a class I carcinogen by the World Health Organization [4]. During the chronic inflammation induced by *H*. *pylori* infection and the subsequent carcinogenesis, various factors, including bacterial, host, and environmental factors, interact to facilitate damage repair. Altered cell proliferation, apoptosis, and some epigenetic modifications to the tumor suppressor genes might occur, which could eventually lead to inflammation associated oncogenesis [2, 3]. Most patients with early-stage gastric cancer are asymptomatic and, therefore, diagnosis is frequently made when disease is at an advanced stage [2]. Patients with advanced GC are, in general, treated with surgery and/or chemotherapy with country-specific guidelines, but relapse and metastasis are common [5, 6]. The current Union International Committee on Cancer (UICC) or the American Joint Committee on Cancer (AJCC) TNM stage system has shown valuable but insufficient estimation for subsets of GC and prediction for prognosis patients [7–9]. Generally, late diagnosis and varied presentations of disease, as well as a general lack of effective therapies to combat disease heterogeneity, are major contributors to the high mortality rate of GC [6]. Thus, there is an urgent need to identify diagnostic, prognostic, and predictive biomarkers to improve patients’ outcomes.

Epigenetic hallmarks along with genetic aberrations have been identified in different subgroups of GC. Accumulating evidence suggests that epigenetic abnormalities in GC are not mere bystander events, but rather promote carcinogenesis through active mechanisms [6]. To date, aberrant DNA methylation is the most extensively studied deregulated epigenetic mechanism in GC [10]. For example, known tumor suppressors or tumor-related genes (p16, RUNX3, MLH1, CDH1, etc.) are silenced by promoter methylation in GC and its precancerous lesions [11]. Generally, aberrant DNA methylation in cancer is classified into two categories: global DNA hypomethylation and regional hypermethylation. Global DNA hypomethylation occurs at CpG dinucleotides, especially in repetitive sequences, which are typically methylated in normal tissues [12, 13]. The latter type of DNA methylation, regional hypermethylation, is relatively more studied in carcinogenesis [14, 15]. Regional hypermethylation occurs preferentially at promoter CpG islands and leads to gene inactivation in the absence of changes to genetic sequence [15].

The Cancer Genome Atlas (TCGA) project demonstrated both genetic and epigenetic profiling for 33 types of human cancer [16]. Based on the multiple platforms utilized within TCGA, it is possible to perform analyses integrating data from multiple sources including transcriptome, methylome, and clinical outcome to explore specific events that are most likely to contribute to oncogenic processes and to identify potential biomarkers associated with patients’ survival. In this study, we performed an integrative analysis to identify the epigenetic changes that may play key role in the initiation and progression of GC, based on which we developed a DNA methylation signature consisting of ten gene members (SCNN1B, NFE2L3, CLDN2, RBPMS2, JPH2, GBP6, COL4A5, SMKR1, PPP1R14A, and ARL4D) to improve survival prediction of GC.

## Methods

### Data acquisition and preprocessing

Level 3 DNA methylation data of GC samples evaluated on the Illumina Infinium HumanMethylation450 platform (450K array) which assesses 482,421 CpG sites throughout the genome were downloaded from the TCGA data portal (https://portal.gdc.cancer.gov/) using the TCGA-Assembler *DownloadMethylationData* function [17]. These data consist of pre-processed data via TCGA pipelines in the form of β values, which are a ratio between methylated probe intensities and total probe intensities. Probe-level data were condensed to a summary beta value for each gene using the *Methylation450*_*single*_*value* function in TCGA-Assembler, which calculates the average methylation value for all CpG sites associated with a gene [18]. We obtained 397 samples of DNA methylation, including 395 gastric adenocarcinoma samples and two normal samples from the methylation data. Methylation data were normalized using limma R package. Level 3 RNA-seq data and clinical information were also retrieved from the TCGA data portal. Among 407 cases of transcriptome profiles, 32 cases were obtained from tumor adjacent tissues, while the remaining 375 cases were GC tissues. The transcriptome data were normalized and log2 transformed with the functions of *DEGList* and *calcNormFactors* in edgeR package [19]. The clinical data were preprocessed by removal of samples without survival status and patients with survival time less than 30 days were also excluded because they might die of non-cancer-related diseases [20]. Above data were available with no restrictions for research, and this study was performed under the guidelines of TCGA.

### Identification of methylation-related differentially expressed genes in GC

First, to acquire differentially expressed genes (DEGs) in GC, the transcriptome data were analyzed using the edgeR package with the *exactTest* function, and a cutoff with false discovery rate (FDR) adjusted *P* < 0.01 and |log_2_FC| ≥ 2 was considered as statistically significant. Next, we explored the association between gene expression and DNA methylation of DEGs in tumor samples. We filtered out tumor samples and DEGs where either gene expression or DNA methylation data was unavailable and Pearson coefficient between gene expression and average methylation level (β value) was calculated. Pearson coefficient < − 0.3 with *P* < 0.05 was set as the criterion for methylation related DEGs (mrDEGs) identification. Heatmap was plotted using pheatmap R package.

### Pathway enrichment analysis of mrDEGs

In order to explore the potential role of the mrDEGs in the initiation and progression of GC, the identified mrDEGs were divided into upregulated and downregulated groups. Gene enrichment analysis for each group was carried out using Metascape, a free online tool for gene annotation (http://metascape.org) [21]. The correction network of the enriched terms was presented in Cytoscape [22].

### Survival model construction process

Prognostic data were created on the methylation matrix of mrDEGS and matched follow-up data. Univariate Cox regression analysis was performed to identify the mrDEGs with prognostic value based on their methylation β value. mrDEGs which were identified significantly associated with overall survival (OS) in the univariate Cox regression analysis (*P* < 0.01) were subjected to the multivariate Cox regression analysis to construct a best fitting prognostic model and a risk score formula was then established by including each of these selected genes, weighted by their estimated regression coefficients in the multivariate Cox regression analysis. Patients were classified into high or low risk groups with the cutoff of the median risk score. We applied the bi-level selection using both forward and backward likelihood ratio tests in the multivariate Cox regression analysis with the Akaike information criterion (AIC) as a stopping rule [23]. Bi-level selection is motivated by the fact that some genes within a gene set may be unrelated to the phenotype of interest, although the gene set as a whole is involved in the biological process [24]. Applying this method to prognostic gene exploration for specific subtypes or stages of a disease, we may screen out gene signatures whose members have subtle individual effects but their coordinated effects are significant when taken together [24]. These steps in the multivariate Cox regression analysis were executed by the survival R package with the function of *coxph*. Apart from model calibration (predicted risk reliability which is indicated by AIC), the discrimination performance indicating the prognostic model’s ability to separate outcome categories was evaluated by Harrell’s concordance index (C-index) with 95% confidence intervals (CIs): values range from 0.5 (classification by 1/2 probability) to 1.0 (perfect prediction) [23]. C-index was calculated using the survcomp R package.

### Validation experiments in gastric cell lines

Quantitative real-time PCR (qPCR), methylation-specific PCR (MSP), and bisulfite sequencing (BSSQ) were used to verify that the expression of certain individual genes in the identified prognostic signature were indeed regulated by DNA methylation. A panel of seven GC cell lines (NUGC3, SNU5, SNU16, NCI-N87, AGS, MGC803, and BGC823) and one gastric epithelial cell line (GES1) were included. All gastric cell lines were preserved in our institute (The First Medical Centre, Chinese PLA General Hospital, Beijing, China). All cell lines were cultured in RPMI 1640 supplemented with 10% fetal bovine serum and 1% penicillin/streptomycin. 5-Aza-2′-deoxycytidine (5-aza, Sigma, St. Louis, MO) treatment (2 μM for 96 h) to these gastric cell lines, RNA isolation, and first strain cDNA synthesis were performed as previously described [25]. qPCR was performed on the StepOnePlus Real-Time PCR System (Applied Biosystems, Foster City, CA) by monitoring the fluorescence of SYBR Green (TaKaRa Bio Inc, Dalian, China) binding to double-stranded DNA. The settings for the PCR thermal were as follows: initial denaturation at 95 °C for 30 s, followed by 40 amplification cycles of 95 °C for 5 s, 60 °C for 15 s, and 72 °C for 15 s. A dissociation analysis was performed at the end of each PCR reaction to ensure its specificity. Each PCR was run in triplicate and repeated three times. For quantification of gene expression changes, the 2^-ΔΔCt^ method [26] was used to calculate relative fold changes normalized against the GAPDH gene. The results were presented as fold changes versus the gene expression level of GES1 cell line without treatment of 5-aza. Primers for qPCR were listed in Additional file 1: Table S1. Genomic DNA was prepared by the proteinase K method. Bisulfite treatment, MSP, and BSSQ were carried out as previously described [27, 28]. CpG island prediction and design of MSP and BSSQ primers were performed using Methyl Primer Express software v1.0 (Thermo Fisher Scientific, Waltham, MA) according to genomic sequence around the transcriptional start site (TSS). BSSQ products were amplified by primers flanking the targeted regions which includes MSP products. The primers for MSP and BSSQ were listed in Additional file 1: Table S1.

### Statistical analysis

Survival differences between the high-risk and low-risk groups were assessed by the Kaplan-Meier estimate, and compared using the log-rank test. The relativity between risk score and clinical factors was analyzed using the chi-square test or Fisher’s exact test. To test whether the risk score derived from the identified DNA methylation signature was independent of patients’ cancer recurrence and TNM stage, multivariate Cox regression and data stratification analysis were performed. We also performed receiver operating characteristic (ROC) analysis to compare the sensitivity and specificity of the survival prediction based on the risk score and TNM stage. Hazard ratios (HR) and 95% CIs were calculated. Kaplan-Meier curve was executed by GraphPad Prism 6 (GraphPad Software, San Diego, CA), while other statistical tests were conducted by R 3.6.0 using the corresponding R package mentioned above.

## Results

### Determining mrDEGs in GC

**Fig. 1:**
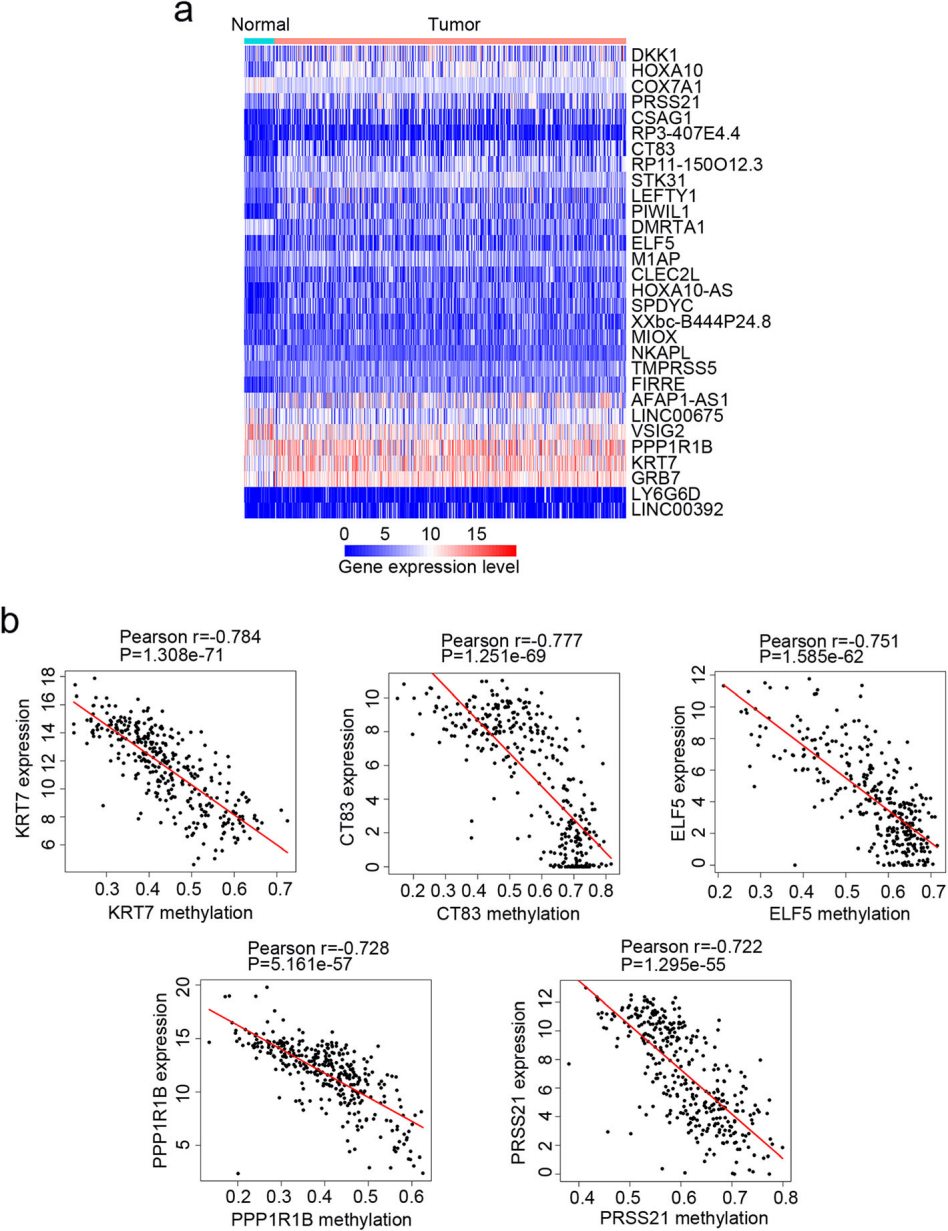
Determining methylation related differentially expressed genes (mrDEGs) in gastric cancer (GC). **a** The expression profile of the most significant 30 mrDEGs between normal (*N* = 32) and GC samples (*N* = 375). **b** The association between gene expression and DNA methylation of the top 5 mrDEGs in GC samples (*N* = 338) whose expression and DNA methylation data were both available

First, we set to identify DEGs between 375 tumor samples and 32 normal samples based on the RNA-seq data of GC. According to the screening criteria (FDR < 0.01 and |log_2_FC| ≥ 2), a total of 3506 significant DEGs were obtained. There were 2210 genes upregulated and 1296 genes downregulated. Next, we explored the association between the mRNA expression and DNA methylation to identify methylation-related DEGs (mrDEGs). We filtered out tumor samples and DEGs where either gene expression or DNA methylation data was unavailable, and 338 tumor samples with 2118 DEGs passed the filter criterion. Then Pearson coefficient between gene expression and average methylation level (β value) for each of the 2118 DEGs was calculated. Totally, 340 DEGs were identified as mrDEGs (Pearson coefficient < − 0.3 and *P* < 0.05; listed in Additional file 2: Table S2). The expression profile of the most significant 30 mrDEGs was shown in Fig. 1a, and the association between gene expression and DNA methylation of the top 5 mrDEGs was shown in Fig. 1b.

### Assessment of relevant biological processes and pathways of mrDEGs

**Fig. 2:**
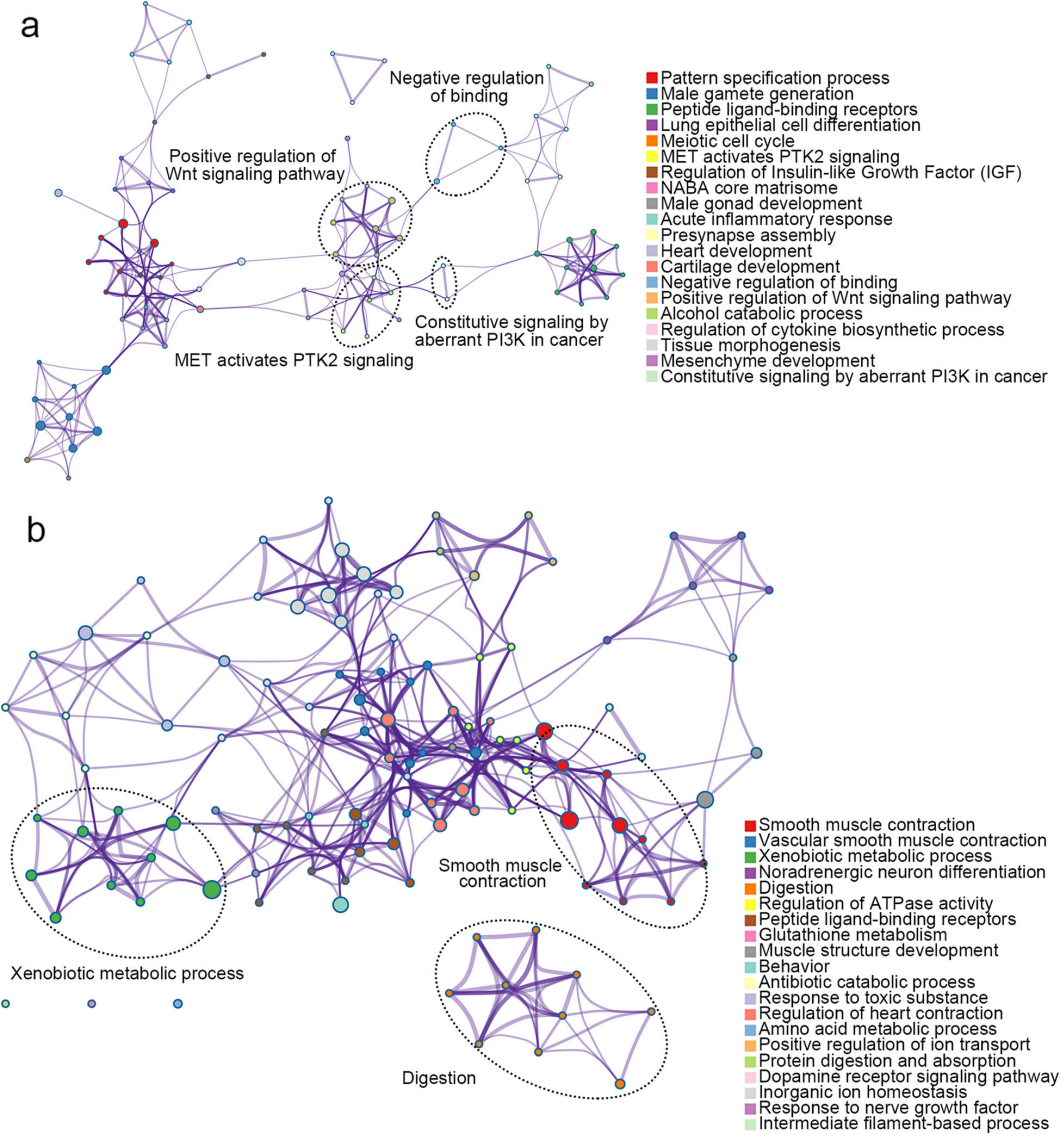
Pathway enrichment analysis of the upregulated and downregulated methylation related differentially expressed genes (mrDEGs) in gastric cancer. **a** The pathway enrichment results of the upregulated mrDEGs. **b** The pathway enrichment results of the downregulated mrDEGs. Each node represents one enriched term. Node size is proportional to the total number of genes within each gene set. Proportion of shared genes between gene sets is represented as the thickness of the line between nodes

To explore the potential role of mrDEGs in the development and progression of GC, the identified mrDEGs were divided into upregulated group (160 mrDEGs) and downregulated group (180 mrDEGs), and Metascape, a free online tool for gene annotation, was used to perform pathway enrichment analysis for each group, respectively. The results showed that the upregulated mrDEGs were enriched in several cancer-related pathways, such as MET activates PTK2 signaling, PI3K signaling, negative regulation of binding, and positive regulation of Wnt signaling pathway (Fig. 2a). Interestingly, KCNMA1, a critical tumor suppressor in GC has been shown to be inactivated by promoter region hypermethylation, and the anti-tumor effect of KCNMA1 is mediated through suppressing the expression of PTK2 [29]. In contrast, the downregulated mrDEGs were mainly enriched in pathways associated with digestive system process, muscle system process, and metabolic process (Fig. 2b). Metabolic reprogramming is considered as a hallmark of cancer [30, 31] and epigenetic-metabolomic interplay plays a critical role in tumorigenesis [32]. For example, epigenome modulation of xenobiotic detoxification pathways reportedly controls predisposition to carcinogen 7,12-dimethylbenz(a)anthracene (DMBA)-induced breast cancer development and progression [33]. Collectively, above findings suggested that the mrDEGs screened in our study are involved in the biological processes of the development and progression of GC.

### Identification of prognostic mrDEGs

After preprocessing of the methylation and clinical data, there were 363 GC patients with both methylation and adequate follow up (survival time no less than 30 days) data, based on which we set to develop a prognostic model for OS prediction. The clinicopathological features of these 363 GC patients for survival model construction were summarized in Additional file 3: Table S3. First, univariate Cox regression analysis was performed on these 363 patients to identify certain prognosis-related mrDEGs according to their methylation β value. We identified a set of 38 mrDEGs whose *P* values were less than 0.01. Those 38 mrDEGs were further subjected to multivariable Cox regression analysis to construct a best fitting prognostic model using the AIC as the indicator for model fitness. Finally, a DNA methylation signature consisting of ten mrDEGs was developed as a prognostic model for GC patients. The identified DNA methylation signature included five gene members (SMKR1, NFE2L3, SCNN1B, ARL4D, and PPP1R14A) with statistically non-significant *P* value (Table 1), but the overall effect was significant and it represented the best fitting prognostic signature with the lowest AIC indicating the most excellent model fitness (global *P* value [log rank] = 6.316e-9, AIC = 1226.66). The C-index of the identified DNA methylation signature was 0.713 (95% CI = 0.668–0.758, *P* = 1.996e-20) suggesting favorable discrimination ability. Among these ten mrDEGs, positive coefficients indicated that the higher methylation levels of four genes (SCNN1B, NFE2L3 and CLDN2, RBPMS2) were associated with shorter survival (risky genes based on methylation level). The negative coefficients for the remaining six genes (JPH2, GBP6, COL4A5, SMKR1, PPP1R14A, and ARL4D) indicated that their higher levels of methylation were associated with longer survival (protective genes based on methylation level). The detailed information of the ten prognostic mrDEGs was shown in Table 1.

**Table 1:** Ten individual genes of the DNA methylation signature associated with overall survival of gastric cancer patients

Gene symbol	Gene name	Chr	Coefficient	*P* value	Associated with DNA methylation
CLDN2	Claudin 2	Xq22.3	4.513	0.044	NR
SMKR1	Small lysine rich protein 1	7q32.1	− 2.918	0.079	NR
NFE2L3	Nuclear factor, erythroid 2 like 3	7p15.2	4.152	0.102	BC [34]
SCNN1B	Sodium channel epithelial 1 beta subunit	16p12.2	2.542	0.102	GC [35] and CCRCC [36, 37]
RBPMS2	RNA binding protein, mRNA processing factor 2	15q22.31	4.765	0.044	NR
JPH2	Junctophilin 2	20q13.12	− 8.525	0.006	NR
COL4A5	Collagen type IV alpha 5 chain	Xq22.3	− 4.225	0.001	CRC [38]
ARL4D	ADP ribosylation factor like GTPase 4D	17q21.31	− 2.367	0.110	NR
GBP6	Guanylate binding protein family member 6	1p22.2	− 7.193	0.000	NR
PPP1R14A	Protein phosphatase 1 regulatory inhibitor subunit 14A	19q13.2	− 2.845	0.066	CRC [39] and ESSC [40]

*NR* not reported, *BC* breast cancer, *GC* gastric cancer, *CCRCC* clear cell renal cell carcinoma, *CRC* colorectal cancer, *ESSC* esophageal squamous cell carcinoma

### The DNA methylation signature for OS prediction of GC patients

**Fig. 3:**
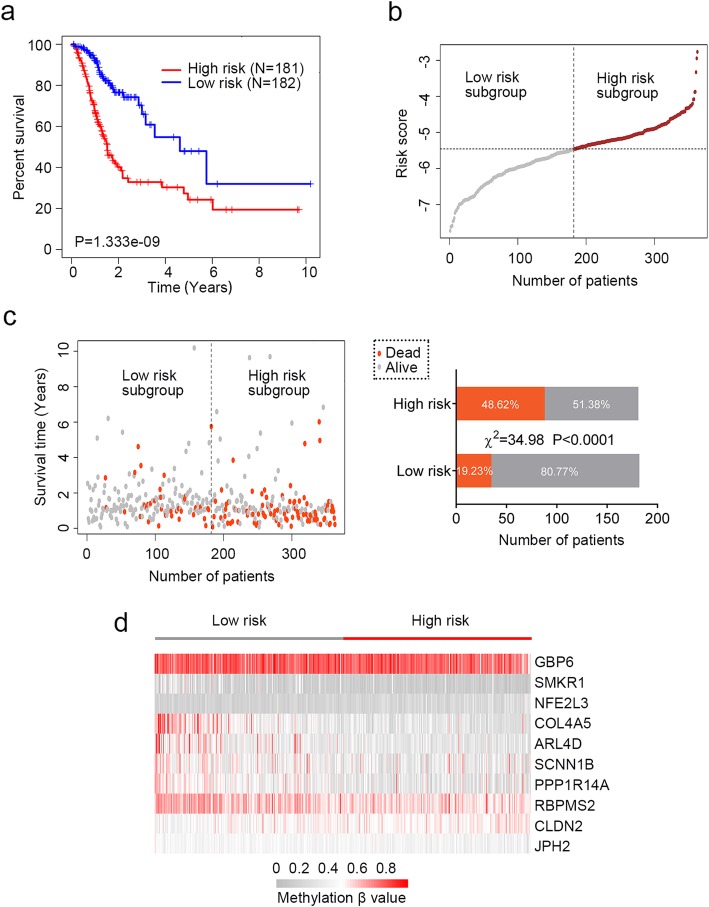
The DNA methylation signature for overall survival (OS) prediction of gastric cancer (GC) patients. **a** Kaplan-Meier estimate of the OS using the identified DNA methylation signature. GC patients were divided into low-risk (*N* = 181) or high-risk (*N* = 182) subgroup based on the median of risk score. The difference between the two curves was determined by the two-side log-rank test. **b** The distribution of risk score derived from the DNA methylation signature. **c** The distribution of GC patients’ survival status. The difference between the low-risk and high-risk subgroup was determined by chi-square test. **d** The methylation β value profile of the identified DNA methylation signature in the high-risk and low-risk subgroups

**Fig. 4:**
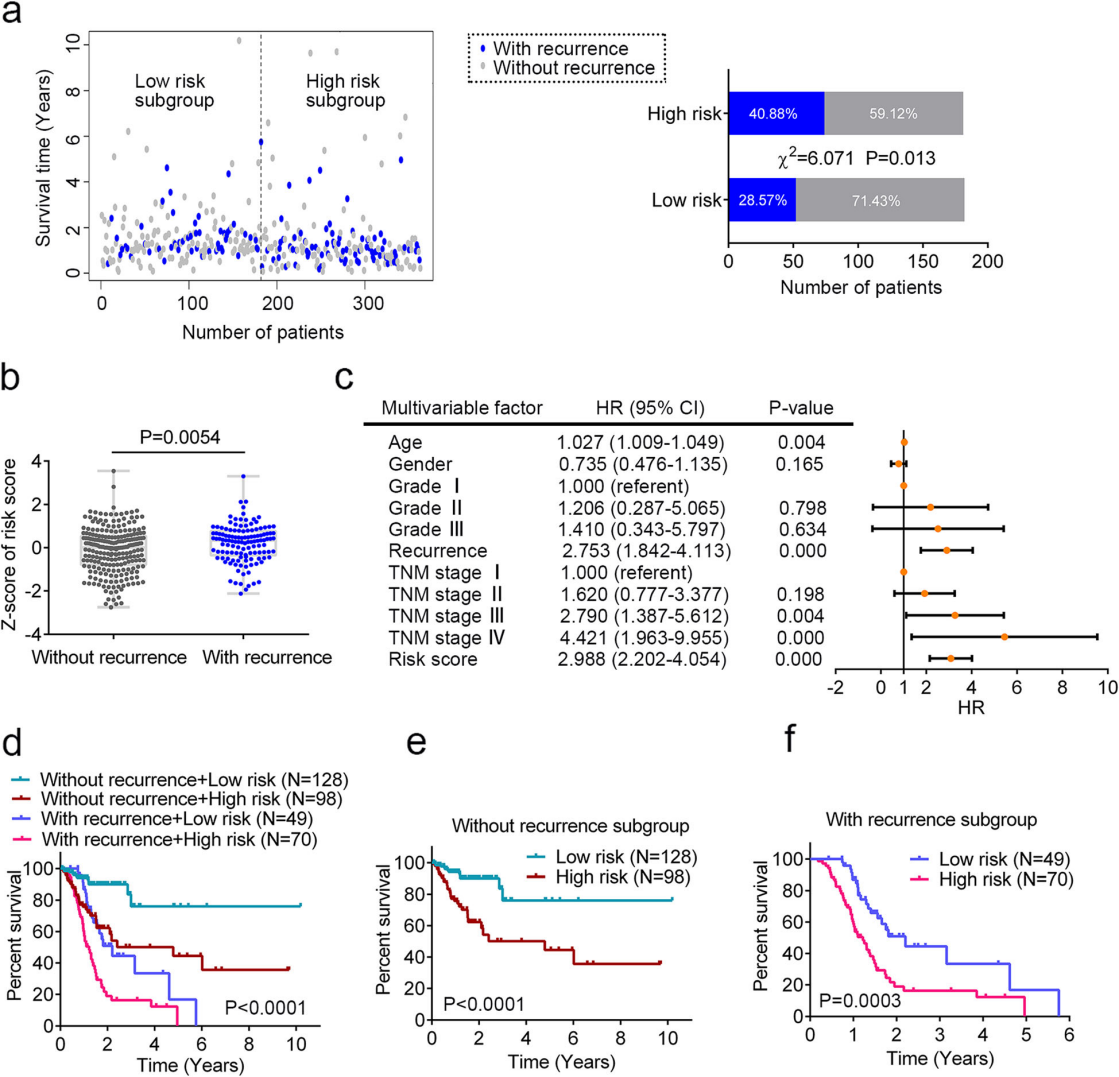
The DNA methylation signature is associated with cancer recurrence. **a** The distribution of cancer recurrence status in the high-risk and low-risk subgroup. The difference between the two subgroups was determined by chi-square test. **b** Box plot of risk score of patients with or without cancer recurrence. *T* test was used to determine the significance of the comparison. **c** The multivariate Cox regression analysis performed on 345 gastric cancer (GC) patients that contained age, gender, tumor grade, cancer recurrence, TNM stage, and risk score as covariates. Risk score and age were evaluated as continuous variables, and gender, tumor grade, cancer recurrence, and TNM stage were evaluated as category variables. Orange solid dots represent the hazard ratio (HR) of death and open-ended horizontal lines represent the 95% confidence intervals (CIs). All *P* values were calculated using Cox proportional hazards analysis. **d** Kaplan-Meier estimate of the overall survival of the entire set GC patients (*N* = 345) using the DNA methylation signature. GC patients were stratified by cancer recurrence status and the high-risk or low-risk subgroup of patients was determined on the basis of the median risk score. **e** Kaplan-Meier curves for GC patients without cancer recurrence (*N* = 226). **f** Kaplan-Meier curves for patients with cancer recurrence (*N* = 119). The differences between the survival curves were determined by the two-sided log-rank test

A risk-score formula was created based on the methylation β values of these ten mrDEGs for OS prediction, as follows: risk score = (4.513* methylation β value of CLDN2) + (− 2.918* methylation β value of SMKR1) + (4.152* methylation β value of NFE2L3) + (2.542* methylation β value of SCNN1B) + (4.765* methylation β value of RBPMS2) + (− 8.525* methylation β value of JPH2) + (− 4.225* methylation β value of COL4A5) + (− 2.367* methylation β value of ARL4D) + (− 7.193* methylation β value of GBP6) + (− 2.845* methylation β value of PPP1R14A). We then calculated the methylation-related risk score for each GC patient, and ranked them according to their risk scores. As such, patients were divided into a high-risk group (*n* = 181) or a low-risk group (*n* = 182) using the median risk score as the cutoff point. Patients in the high-risk group had significantly shorter median OS than those in the low-risk group (log-rank test *P* < 0.0001) (Fig. 3a). Moreover, the association of the risk score with OS was also significant when it was evaluated as a continuous variable in the multivariate Cox regression analysis (Fig. 4c). The distribution of risk score, survival status, and methylation β value in GC patients were also profiled (Fig. 3b–d). Patients in the high-risk group tended to display high methylation levels of risky mrDEGs (SCNN1B, NFE2L3 and CLDN2, RBPMS2), whereas patients in the low-risk group tended to display high methylation levels of protective mrDEGs (JPH2, GBP6, COL4A5, SMKR1, PPP1R14A, and ARL4D).

### The DNA methylation signature is associated with cancer recurrence

Next, we analyzed the relativity between clinicopathological features and the risk score derived from the identified DNA methylation signature. The results showed that GC patients in high-risk group were more likely to have cancer recurrence (Table 2 and Fig. 4a; χ^2^ = 6.071, *P* = 0.013). We also evaluated the risk score as a continuous variable and compared it between patients with recurrence and without recurrence. Patients with recurrence tended to have higher risk score than patients without recurrence (Fig. 4b, *P* = 0.0054). Interestingly, the risk score was also significantly associated with patients’ gender (Table 2, *P* < 0.001). Aberrant methylation of genes on X chromosomes has been proven to be involved in carcinogenesis and it is associated with gender differences in cancer risk [41–43]. Two individual genes (CLDN2 and COL4A5) of the DNA methylation signature are located on the X chromosome; therefore, we plausibly hypothesized that the abnormal methylation of the prognostic signature could show gender disparity.

**Table 2:** Correlations between clinical characteristics and risk score derived from the DNA methylation signature

Variable	*N*	High risk	Low risk	*P* value
Age (years)	363			0.051
≥ 60	185	93	92	
< 60	96	52	44	
Gender	363			0.000*
Male	241	136	105	
Female	122	45	77	
Race	330			0.106
White	235	108	127	
Non-white	95	53	42	
Family history	315			1.000
Yes	17	9	8	
No	298	150	148	
*H*. *pylori* infection	177			1.000
Positive	20	9	11	
Negative	157	70	87	
Tumor location	362			0.183
Cardia region (Cardia + GEJ)	91	51	40	
Non-cardia region	271	130	141	
Grade	354			0.380
G3	221	112	109	
G1 + G2	133	61	72	
T stage	363			0.562
T3+T4	274	139	135	
T1+T2	89	42	47	
N stage	355			0.463
N1 + N2 + N3	243	123	120	
N0	112	52	60	
M stage	350			0.176
M1	21	14	7	
M0	329	161	168	
TNM stage	349			0.954
III + IV	186	93	93	
I + II	163	82	81	
Recurrence	363			0.014*
Yes	126	74	52	
No	237	107	130	

*P* values were acquired by chi-square test or Fisher’s test. *P value < 0.05 was considered statistically significant. *GEJ* gastro-esophageal junction

Since cancer recurrence could strongly affect patients’ OS, we tested whether the prognostic value of the identified DNA methylation signature was independent of cancer recurrence. For this, we conducted multivariable Cox regression and stratification analysis. The multivariable Cox regression analysis was performed on 345 patients that contained age, gender, tumor grade, cancer recurrence, TNM stage, and risk score as covariates. Eighteen cases of GC patients were not included because of incomplete record of tumor grade and TNM stage. The results showed that the risk score (HR = 2.988, 95% CI = 2.202–4.054, *P* < 0.0001) and cancer recurrence (HR = 2.753, 95% CI = 1.842–4.113, *P* < 0.0001) were both independent prognostic factors (Fig. 4c). Data stratification analysis was then performed where these patients were stratified into cancer recurrence subgroup and non-recurrence subgroup. The stratification analysis showed that the DNA methylation signature could identify patients with different prognoses regardless of the disease relapse status (Fig. 4d). For instance, among the patients without cancer recurrence, the risk score could further subdivide them into those likely to have longer versus shorter survival (log-rank test *P* < 0.0001, Fig. 4e). Similarly, among those with recurrence, the risk score could also subdivide patients into two subgroups with significantly disparate survival (log-rank test *P* = 0.0003, Fig. 4f).

### Prognostic value of the DNA methylation signature is independent of TNM stage

**Fig. 5:**
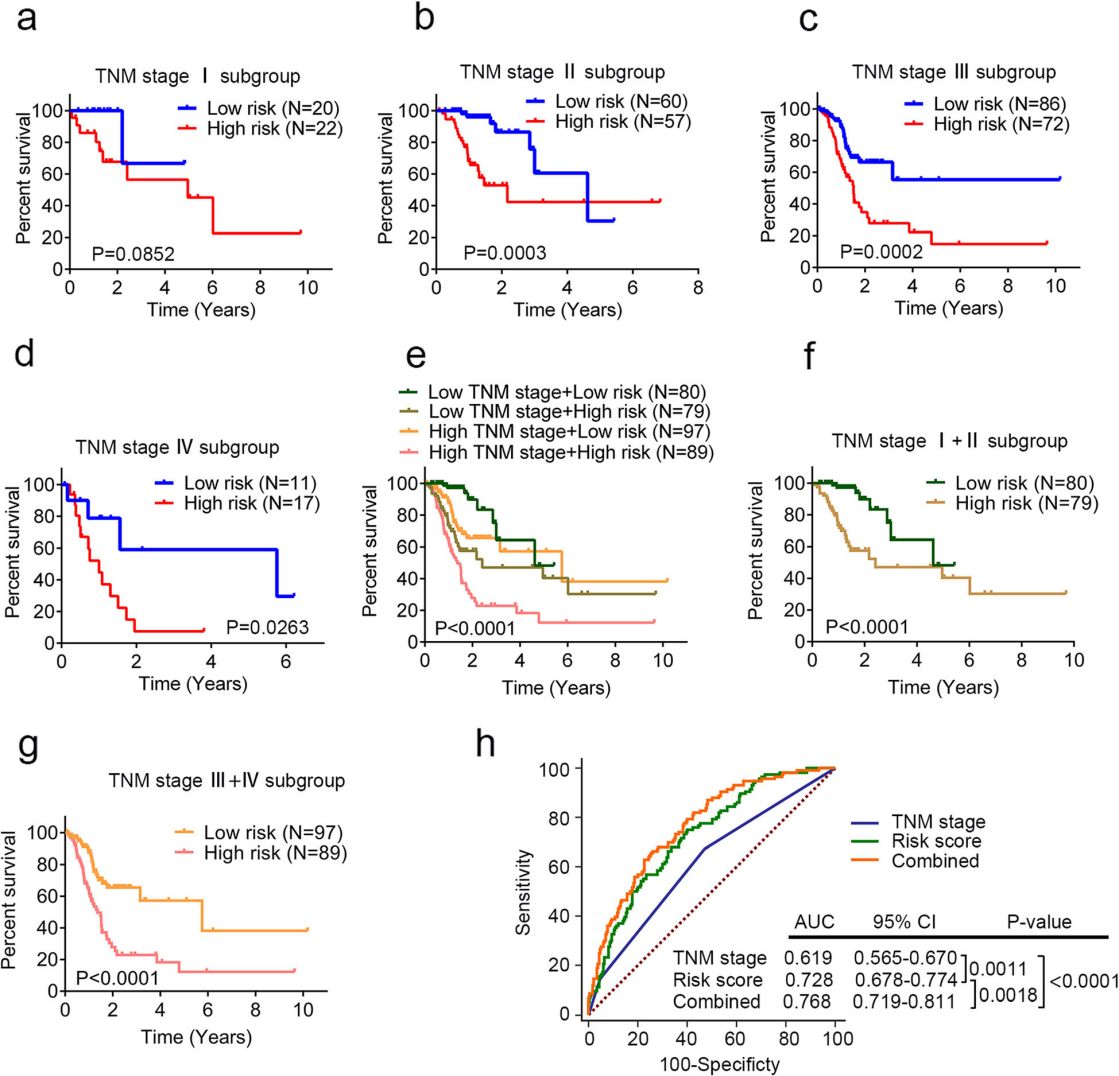
Prognostic value of the DNA methylation signature is independent of TNM stage. **a** Kaplan-Meier curves for patients with TNM stage I (*N* = 42). Gastric cancer (GC) patients were stratified by TNM stage (I, II, III, and IV) and the high-risk or low-risk subgroup of patients was determined on the basis of the median risk score. **b** Kaplan-Meier curves for patients with TNM stage II (*N* = 117). **c** Kaplan-Meier curves for patients with TNM stage III (*N* = 158). **d** Kaplan-Meier curves for patients with TNM stage IV (*N* = 28). **e** Kaplan-Meier estimate of the overall survival (OS) of the entire set GC patients (*N* = 345) using the DNA methylation signature. GC patients were stratified by TNM stage (I + II and III + IV). **f** Kaplan-Meier curves for patients with low TNM stage (stage I + II, *N* = 159). **g** Kaplan-Meier curves for patients with high TNM stage (stage III + IV, *N* = 186). The differences between the survival curves were determined by the two-sided log-rank test. **h** Receiver operating characteristic (ROC) analysis of the sensitivity and specificity of OS prediction by the DNA methylation signature risk score, TNM stage, and combination of the two factors. *P* values were obtained from the comparisons of the area under the ROC (AUROC) of DNA methylation signature risk score versus those of TNM stage and DNA methylation risk score combined with TNM stage

The UICC/AJCC TNM system is currently the most prevalent criterion for tumor staging, providing a useful bench mark for selection of treatment modalities and survival prediction [44, 45]. Thus, we further tested whether the prognostic value of the identified DNA methylation signature was independent of TNM stage. The multivariable Cox regression analysis showed that both risk score and TNM stage were independent prognostic factors (Fig. 4c). The stratification analysis suggested that the DNA methylation signature could identify patients with different prognoses in each TNM stage subgroup (Fig. 5a–d) despite that the *P* value was not significant in stage I (log-rank test *P* = 0.0852). This might be attributed to the small sample size to draw any reliable conclusions. Then we combined low TNM stage (I and II) and high TNM stage (III and IV), respectively; the risk score could still identify patients with different prognoses in each subgroup and the *P* value was significant (Fig. 5e–g).

Furthermore, we performed ROC analysis to compare the sensitivity and specificity of OS prediction among the identified DNA methylation risk score model, TNM stage, and combination of these two factors. The areas under receiver operating characteristic (AUROCs) were assessed and compared. As shown in Fig. 5h, the AUCROC of the identified DNA methylation signature was significantly greater than TNM stage (0.728 versus 0.619, *P* = 0.0011). Additionally, the AUCROC of risk score combined with TNM stage was significantly superior than TNM stage (0.768 versus 0.619, *P* < 0.0001) or the risk score (0.768 versus 0.728, *P* = 0.0018) alone. These results indicated that the combination of the identified DNA methylation signature and TNM stage may help improve OS prediction in patients with GC.

### The expression of PPP1R14A and SCNN1B, two individual genes of the identified prognostic signature, are regulated by promoter region methylation

**Fig. 6:**
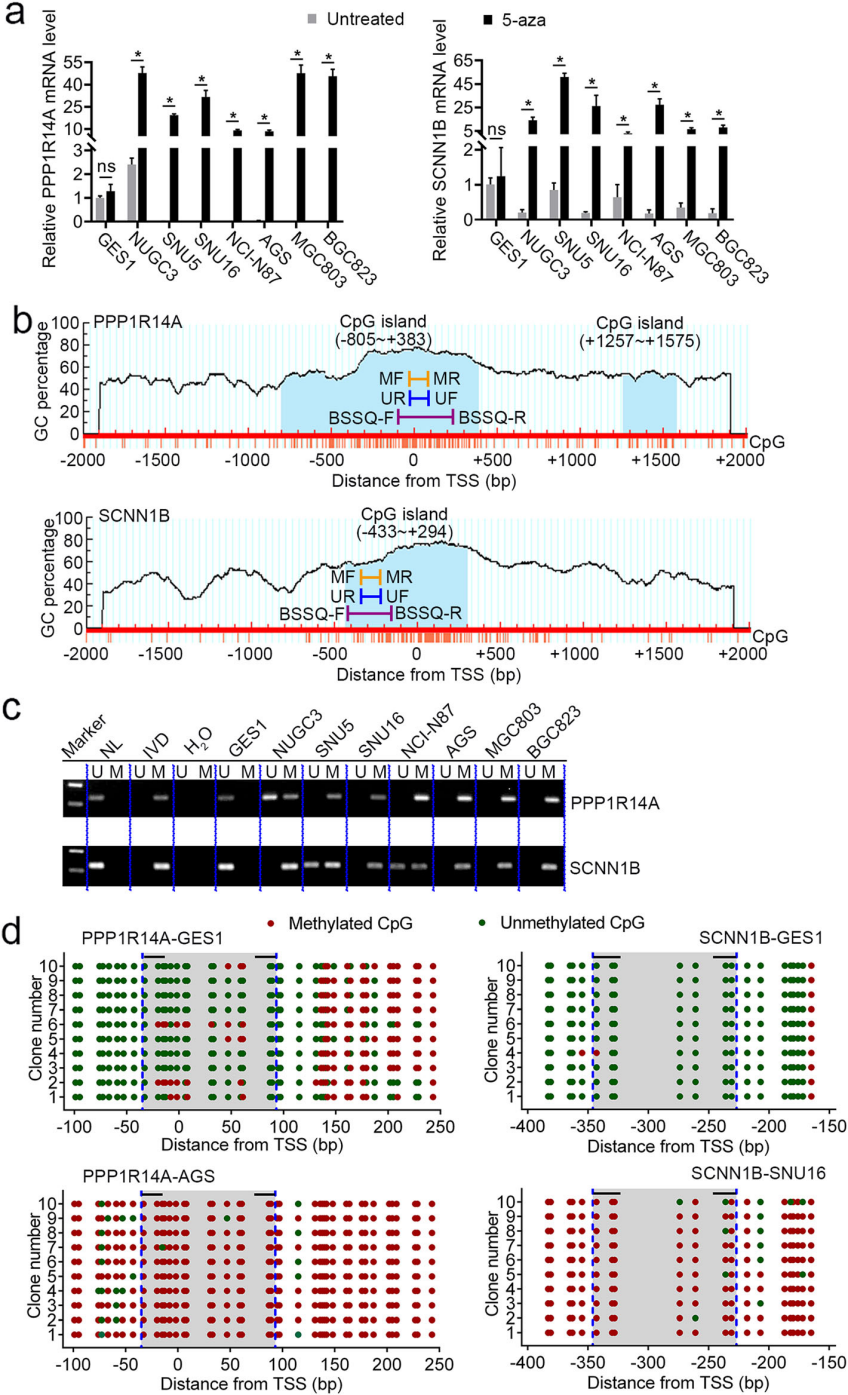
The expression of PPP1R14A and SCNN1B, two individual genes of the identified prognostic signature, are regulated by promoter region methylation. **a** Expression levels of PPP1R14A and SCNN1B without (−) or with (+) 5-aza treatment were analyzed by qPCR in eight gastric cell lines (GES1, NUGC3, SNU5, SNU16, NCI-N87, AGS, MGC803, and BGC823). **b** Schematic diagrams of CpG islands in the promoter region of PPP1R14A and SCNN1B. *MF* methylation forward primer, *MR* methylation reverse primer, *UF* unmethylation forward primer, *UR* unmethylation reverse primer, *BSSQ*-*F* bisulfite sequencing forward primer, *BSSQ*-*R* bisulfite sequencing reverse primer. **c** Methylation status of PPP1R14A and SCNN1B was detected by methylation specific PCR (MSP) in gastric cell lines. *IVD* in vitro methylated DNA, *NL* normal lymphocyte DNA, *M* methylated alleles, *U* unmethylated alleles. **d** Bisulfide sequencing (BSSQ) of PPP1R14A was performed in GES1 and AGS cell lines. For SCNN1B, GES1 and SNU16 cell lines were analyzed. Red solid dots represent methylated CpG sites, and green solid dots denote unmethylated CpG sites. The horizontal black bar demarcates the primers of MSP, which are included in the region of BSSQ

The prognostic methylation signature consists of ten gene members, some of which have been reported to be dysregulated in cancer. For example, PPP1R14A was previously described to act as an oncoprotein in the merlin pathway [46], while SCNN1B is classified as a member channel with tumor-suppressive effect [35]. However, the expression of these two genes and the underlying regulatory mechanisms in GC have not been fully uncovered and thus deserve further investigation. First, we examined the level of PPP1R14A mRNA expression using qPCR in the eight gastric cell lines. PPP1R14A was highly expressed in GES1 and NUGC3 cells, whereas loss of PPP1R14A expression was found in SNU5, SNU16, NCI-N87, AGS, MGC803, and BGC823 cells (Fig. 6a, left panel). CpG islands situated in the PPP1R14A gene promoter region and the designed MSP and BSSQ primers were shown in Fig. 6b (upper panel). MSP was applied to evaluate its methylation status. Unmethylation and partial methylation of PPP1R14A were found in GES1 and NUGC3 cells with PPP1R14A expression, respectively (Fig. 6c, upper panel). By contrast, complete methylation was detected in the remaining six gastric cell lines with dim expression of PPP1R14A (Fig. 6c, upper panel). The results of qPCR and MSP revealed a negative correlation between PPP1R14A methylation and mRNA expression. To test whether promoter methylation directly contributes to transcriptional silencing of PPP1R14A, the gastric cell lines were treated with 5-aza, a demethylation agent. Restoration of PPP1R14A expression was found in the six methylated cell lines and increased expression of PPP1R14A was detected in partial methylated NUGC3 cells, whereas no significant expression change was observed in unmethylated GES1 cells (Fig. 6a, left panel). We also used BSSQ technique to validate the efficiency of MSP primers and to assess the methylation density of a prolonged genomic sequence in the PPP1R14A promoter region. Consistent with MSP results, the BSSQ analysis revealed methylation and unmethylation of PPP1R14A in AGS and GES1 cells, respectively (Fig. 6d, left panel). Collectively, these results suggested that the expression of PPP1R14A is regulated by promoter region methylation in gastric cell lines. Similarly, the expression of SCNN1B was also proved to be regulated by promoter region methylation (Fig. 6a–d).

## Discussion

Recent researches have well documented the impact of genetic events on the initiation, development, and progression of cancer. However, emerging evidence shows that apart from genetic lesions, alteration to the epigenome is also a fundamental characteristic of nearly all human cancers [47]. Epigenetic changes can be examined at the level of histone modifications, chromatin conformation, or DNA methylation [47, 48]. DNA methylation has been the main focus due to the quantitative nature of DNA methylation assays, and the relative ease of obtaining sufficient genomic DNA compared to chromatin [47]. Pioneering studies identified decreased 5-methylcytosine content in tumors compared to normal tissue [49, 50], further loss of 5-methylcytosine during tumor progression [51], and increased methylation in normally unmethylated CpG islands and promoter regions of a wide variety of genes including tumor suppressors [52, 53], metastasis genes [54, 55], and DNA repair genes [56, 57]. The changes were hypothesized to affect gene expression and chromosomal stability. More recently, aberrant DNA methylation has been associated with biology of GC and can mark the spectrum of disease progression, thus serving as biomarkers for GC diagnosis and prognosis [58]. Additionally, a multitude of cutting-edge tools, for instance, the next-generation sequencing and bioinformatics tools, pave the way for integrated analysis of genetic and epigenetic changes in human cancers [59, 60]. The TCGA project provides valuable data source generated on multiple platforms including transcriptome, methylome, and clinical outcome for us to identify specific events that are most likely to contribute to oncogenic processes and discover potential biomarkers associated with patients’ outcome.

**Fig. 7:**
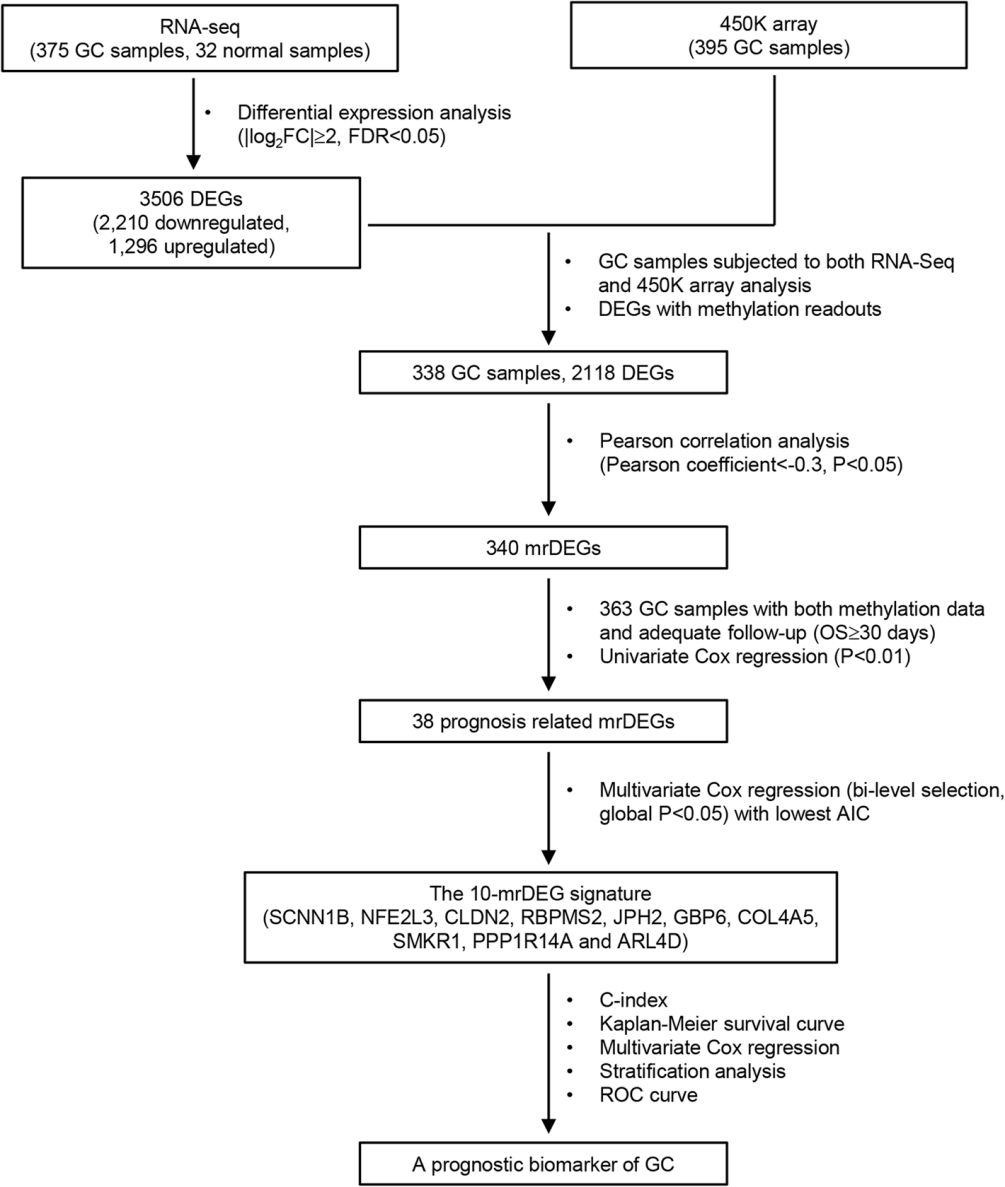
Flowchart showing steps involved in identification of the prognostic DNA methylation signature in gastric cancer

In this study, through mining the multi-source data from TCGA, we performed an integrative analysis to identify the epigenetic changes that may play key role in the development and progression of GC, based on which we developed a DNA methylation signature for prognosis prediction of GC (Fig. 7). We finally identified a set of ten methylation-related genes (SCNN1B, NFE2L3, CLDN2, RBPMS2, JPH2, GBP6, COL4A5, SMKR1, PPP1R14A, and ARL4D) that showed differential expression among the GC patients from TCGA. Such differentiation signified their potential roles in GC. Although some of these genes have been reported to be dysregulated in cancer or other disorders, methylation of these genes or their biological role in GC has not been thoroughly investigated. For example, COL4A5 has been reported to play active role in cell growth and angiogenesis [61]. Loss of COL4A5 expression in colorectal cancer is associated with hypermethylation of its promoter region [38]. In our study, expression of COL4A5 was negatively correlated with DNA methylation in GC and hypermethylation of COL4A5 was associated with prolonged survival. Thus, we infer that COL4A5 may act as an oncogene in GC tumorigenesis and further investigations are greatly needed. Rauscher et al. found that in breast cancer NFE2L3 displays hypermethylation for estrogen receptor (ER)-positive tumors and hypomethylation for ER-negative tumors, and methylation level of its promoter region exhibits an inverse correlation with expression among the cancer samples [34]. In the present study, expression of NFE2L3 was negatively correlated with DNA methylation in GC and hypermethylation of NFE2L3 was associated with shorter OS. Another candidate, SCNN1B, a part of a multiprotein complex consisting of three subunits that control fluid and electrolyte transport across epithelia in diverse organs [62], has been shown to be silenced by promoter methylation in GC [35] and clear cell renal cell cancer [36, 37]. Yun Qian et al. revealed that SCNN1B mRNA expression is silenced by promoter hypermethylation in GC cell lines and primary tumor tissues and high SCNN1B expression is an independent prognostic factor that predicts better survival in a cohort of 245 GC patients [35]. The tumor-suppressive effect of SCNN1B is mediated via degradation of GRP78, a chaperone with oncogenic properties [35]. In line with above findings, hypermethylation of SCNN1B was identified as a risky factor with a positive coefficient in the prognostic DNA methylation signature in the present study. In addition, by conducting qPCR, MSP, and BSSQ analysis in our panel of gastric cell lines, we also found that SCNN1B was unexpressed in most of the GC cell lines examined, whereas normally expressed in the immortalized human gastric mucosa cell line GES1, and its expression was regulated by promoter region methylation. PPP1R14A was reportedly downregulated in the CRC tissue samples while upregulated in CRC cell lines following 5-aza treatment [39]. In our study, we verified that the expression of PPP1R14A was regulated by promoter region methylation in GC using MSP and BSSQ analysis. As for the rest of genes, regulation by DNA methylation has not been reported previously. Our findings suggest that they deserve further investigations to clarify their potential as DNA methylation biomarkers in GC.

By applying the risk score model of DNA methylation signature to GC patients, a clear separation was observed in survival curve between patients in high-risk and low-risk subgroups. There were significant associations between OS and the identified DNA methylation signature, which was evaluated as a category variable (divided by median cutoff) or continuous variable. We also uncovered that the identified DNA methylation signature was associated with cancer recurrence. Furthermore, we performed multivariate Cox regression and stratification analysis, and the results suggested that the prognostic value of the DNA methylation signature was independent of cancer recurrence and TNM stage which are two main prognostic factors in GC. Finally, it was fascinating to find that the identified DNA methylation signature had a stronger predictive power than TNM stage in the ROC analysis. Moreover, when combined with TNM stage, the DNA methylation signature showed even better predictive ability. These results indicate that the combination of the identified DNA methylation signature and TNM stage may help improve OS prediction in patients with GC.

Circulating tumor DNA (ctDNA) consists of extracellular nucleic acid fragments shed into plasma via tumor cell necrosis, apoptosis, and active release of DNA [63]. ctDNA exhibits genetic and epigenetic alterations from its cell of origin and therefore is emerging as a major tool allowing for real-time and dynamic monitoring of molecular changes of tumor in precision medicine. ctDNA-bearing cancer-specific methylation patterns have been investigated as feasible biomarkers in cancers [64]; however, currently there are only a few validated methylation markers available, such as SEPT9 in colorectal cancer [65]. In the present study, considering the differential methylation level of the identified prognostic gene panel between high-risk and low-risk GC patients and its association with cancer recurrence, it has great potential to become a useful candidate methylation marker of ctDNA in precise stratifying GC patients and temporal monitoring cancer recurrence in the future clinical trials.

Although the predictive performance of the identified DNA methylation was quite favorable, the limitations should be acknowledged for our study. First, since insufficient normal samples (two cases) were evaluated for DNA methylation in GC patients from TCGA, we could not identify genes differentially methylated between normal and tumor samples, which would otherwise uncover genes that are more likely to contribute to oncogenic processes of GC by integrating with differential expression analysis. Thus, this methylation signature is useful for determining prognosis of GC only, while it cannot be taken as a diagnostic biomarker. Second, there is no additional dataset for external validation. We searched the Gene Expression Omnibus (https://www.ncbi.nlm.nih.gov/geo/) for methylation datasets with available survival information, but our attempt was futile because of either insufficient sample size or different platforms for methylation detection. Despite of this drawback, however, considering the large amount of GC patients (363 cases) included for the development of the prognostic model, it is more likely to be a significant determinant of survival in GC rather than an accidental feature of methylome noise. Finally, we have limited experimental data and lack information on the regulatory mechanisms and functional roles of the individual genes of the DNA methylation signature. Further experimental studies on these genes are greatly warranted to clarify their potentials as DNA methylation biomarkers for GC.

## Conclusions

This study presents a powerful DNA methylation signature by performing analyses integrating multi-source data including transcriptome, methylome, and clinical outcome of GC patients from TCGA. This innovative DNA methylation signature was associated with cancer recurrence, while it showed independence of cancer recurrence and TNM stage, two main prognostic factors in GC, for survival prediction. Combination of this DNA methylation signature and TNM stage improved OS prediction for GC patients. We verified that two individual genes (PPP1R14A and SCNN1B) of the identified prognostic signature were regulated by promoter region methylation. Further experimental studies are warranted to unveil the regulatory mechanisms and functional roles of all the individual genes of the DNA methylation signature. Also clinical investigations in large GC patient cohorts are greatly needed to validate our findings.

## Supplementary information

**Additional file 1: Table S1**. Primers used in this study.

**Additional file 2: Table S2**. A total of 340 methylation related differentially expression genes were screened in gastric cancer patients from TCGA.

**Additional file 3: Table S3**. Clinical characteristics of 363 gastric cancer patients with both methylation data and adequate follow-up information for prognostic model construction.

## Data Availability

Clinical information, high-throughput sequencing-counts, and DNA methylation data were retrieved from the TCGA data portal, which is a publicly available database.

## Abbreviations

450K array: Illumina Infinium HumanMethylation450
5-aza: 5-aza-2′-deoxycytidine
AIC: Akaike information criterion
AJCC: American Joint Committee on Cancer
AUROC: Area under receiver operating characteristic
BSSQ: Bisulfite sequencing
CI: Confidence interval
C-index: Concordance index
ctDNA: Circulating tumor DNA
DEGs: Differentially expressed genes
ER: Estrogen receptor
FDR: False discovery rate
GC: Gastric cancer
HR: Hazard ratio
mrDEGs: Methylation related differentially expressed genes
MSP: Methylation specific PCR
OS: Overall survival
qPCR: Quantitative real-time PCR
ROC: Receiver operating characteristic
TCGA: The Cancer Genome Atlas
TSS: Transcriptional start site
UICC: Union International Committee on Cancer
