# Supplementary material for: A DNA methylation signature to improve survival prediction of gastric cancer
Source: Clin Epigenetics. 2020 Jan 20;12:15. doi: 10.1186/s13148-020-0807-x (PMC6972030; doi:10.1186/s13148-020-0807-x)
Supplement: Supplementary file 1 — Additional file 1: Table S1. Primers used in this study. [file 13148_2020_807_MOESM1_ESM.docx]

**Table S1.** Primers used in this study.

| Primers | Sequence (5’-3’) |
| --- | --- |
| qPCR-PPP1R14A (F) | AGGCAGACATGCCCGATGAG |
| qPCR-PPP1R14A (R) | TCCTGGATGAAGTCCTCGACAG |
| qPCR-SCNN1B (F) | ATCGGGGTACTCGTGGACA |
| qPCR-SCNN1B (R) | AGTGGCCACAGTTGCAGTTA |
| qPCR-GAPDH (F) | TGCACCACCAACTGCTTAGC |
| qPCR-GAPDH (R) | GGCATGGACTGTGGTCATGAG |
| MSP-PPP1R14A (MF) | CGTAAGAAGTATTCGGGGC |
| MSP-PPP1R14A (MR) | ACCCGCGATAAAAACTACAA |
| MSP-PPP1R14A (UF) | GTTTGTAAGAAGTATTTGGGGT |
| MSP-PPP1R14A (UR) | ACCCACAATAAAAACTACAACTT |
| MSP-SCNN1B (MF) | GAACGGGATAGGTATACGC |
| MSP-SCNN1B (MR) | ATACGCCACGAATATATCCA |
| MSP-SCNN1B (UF) | GGTGAATGGGATAGGTATATGT |
| MSP-SCNN1B (UR) | ACATACACCACAAATATATCCAC |
| BSSQ-PPP1R14A (F) | GGGGAAATTGAGGTTTGA |
| BSSQ-PPP1R14A (R) | CTCCCCRAACTCACCATAC |
| BSSQ-SCNN1B (F) | GAGGATATTGTGTTTGTAGGGG |
| BSSQ-SCNN1B (R) | TTCAAAAACACTAAACRCCC |
