# Supplementary material for: A DNA methylation signature to improve survival prediction of gastric cancer
Source: Clin Epigenetics. 2020 Jan 20;12:15. doi: 10.1186/s13148-020-0807-x (PMC6972030; doi:10.1186/s13148-020-0807-x)
Supplement: Supplementary file 3 — Additional file 3: Table S3. Clinical characteristics of 363 gastric cancer patients with both methylation data and adequate follow-up information for prognostic model construction. [file 13148_2020_807_MOESM3_ESM.docx]

**Table S3.** Clinical characteristics of 363 gastric cancer patients with both methylation data and adequate follow-up information for prognostic model construction.

| **Variable** | **Number of cases (Total = 363)** |
| --- | --- |
| **Age (years)** |  |
| <60/>=60 | 115/248 |
| **Gender** |  |
| Female/Male | 122/241 |
| **Race** |  |
| White/Asian/Black of African American/Native Hawaiian or other pacific islander/NA | 235/82/12/1/33 |
| **Family history** |  |
| No/Yes/NA | 298/17/48 |
| ***H pylori* infection** |  |
| Negative/Positive/NA | 157/20/186 |
| **Tumor location** |  |
| Cardia/GEJ/Antrum/Fundus/NOS/NA | 50/41/131/132/8/1 |
| **Grade** |  |
| G1/G2/G3/Gx | 9/124/221/9 |
| **T stage** |  |
| T1/T2/T3/T4 | 16/73/176/98 |
| **N stage** |  |
| N0/N1/N2/N3/Nx/NA | 112/93/74/76/6/2 |
| **M stage** |  |
| M0/M1/Mx | 329/21/13 |
| **TNM stage** |  |
| Ⅰ/Ⅱ/Ⅲ/Ⅳ/NA | 44/119/160/30/10 |
| **Recurrence** |  |
| No/Yes | 237/126 |

GEJ: Gastro-oesophageal junction; NOS: Not other specified; NA: Not available.
